# Role of STAT3 signaling pathway in breast cancer

**DOI:** 10.1186/s12964-020-0527-z

**Published:** 2020-02-28

**Authors:** Jia-hui Ma, Li Qin, Xia Li

**Affiliations:** 1 Marine College, Shandong University, Wenhua West Rd. 180, Weihai, Shandong 264209 P.R. China; 2 Department of Pathology and Lab Medicine, Institute of Hematology and Blood Diseases Hospital, Chinese Academy of Medical Sciences, Tianjin, China; 3 Tianjin Sino-US Diagnostics Co., Ltd., Tianjin, PR China; 4 School of Pharmaceutical Sciences, Shandong University, Jinan, 250012 China

**Keywords:** STAT3, Breast cancer, Oncogene, Small molecule inhibitors

## Abstract

Breast cancer has grown to be the second leading cause of cancer-related deaths in women. Only a few treatment options are available for breast cancer due to the widespread occurrence of chemoresistance, which emphasizes the need to discover and develop new methods to treat this disease. Signal transducer and activator of transcription 3 (STAT3) is an early tumor diagnostic marker and is known to promote breast cancer malignancy. Recent clinical and preclinical data indicate the involvement of overexpressed and constitutively activated STAT3 in the progression, proliferation, metastasis and chemoresistance of breast cancer. Moreover, new pathways comprised of upstream regulators and downstream targets of STAT3 have been discovered. In addition, small molecule inhibitors targeting STAT3 activation have been found to be efficient for therapeutic treatment of breast cancer. This systematic review discusses the advances in the discovery of the STAT3 pathways and drugs targeting STAT3 in breast cancer.

Video abstract

Video abstract

## Background

**Fig. 1:**
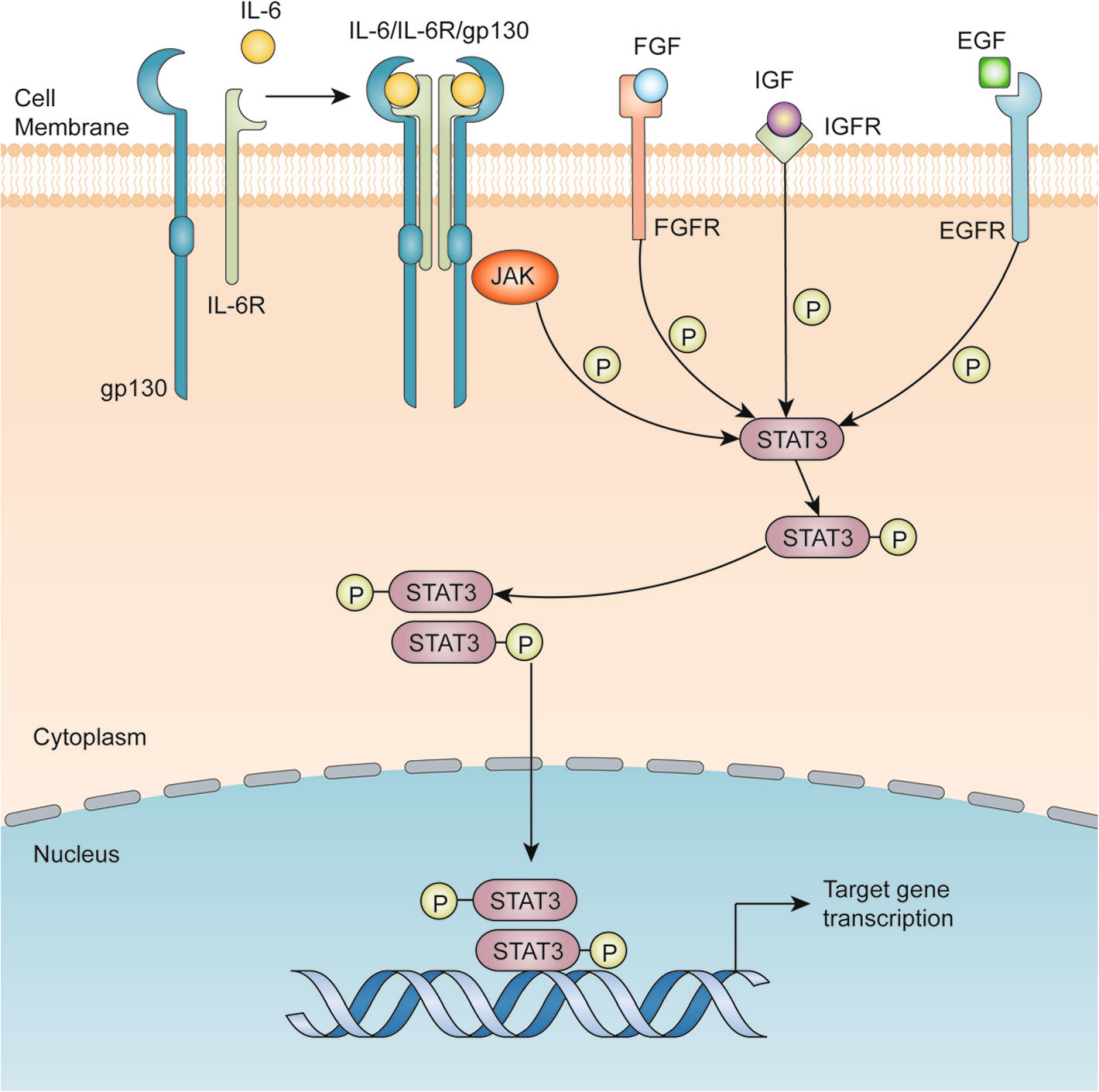
The classical IL-6/STAT3 signaling pathway in cancer cells. IL-6 binds to the membrane-bound IL-6 receptor α (IL-6R) and IL-6 receptor β (also known as gp130). The IL-6/IL-6R/gp130 complex activate the phosphorylation of JAKs, followed by STAT3 phosphorylation and activation. Growth factors, such as FGF, IGF and EGF, can also phosphorylate STAT3 by binding to their cognate membrane receptors. Then, phosphorylated STAT3 forms a homodimer and translocates into the nucleus to bind to the promotor region of target genes and activates target gene transcription

Transcription factors (TFs) are proteins possessing domains that bind to the DNA of promoter or enhancer regions of specific genes. Several TFs are directly involved in the development and progression of breast cancer. One of the most prominent TF families in breast cancer is the signal transducers and activators of transcription (STAT) family, which is comprised of seven structurally similar and highly conserved members, namely, STAT1, STAT2, STAT3, STAT4, STAT5a, STAT5b and STAT6 [1, 2]. In general, these family members contain six common functional domains: an N-terminal domain (NH2) which is called STAT_int now, a coiled-coil domain (CCD), a DNA-binding domain (DBD), a linker domain, an SRC homology 2 domain (SH2) and a transactivation domain (TAD) [3]. Since the discovery of STAT3 in 1994, research has been primarily focused on its close association with cancer progression, proliferation, metastasis and multidrug resistance [4, 5]. Extensive reviews have described the classical STAT3 signaling pathways [6–8]. Here, we present a short overview of the STAT3 signaling pathways as depicted in Fig. 1. Briefly, STAT3 is activated through several cytokines, including interleukin 6 (IL-6) and interleukin 10 (IL-10), and growth factors, including epidermal growth factor (EGF), fibroblast growth factor (FGF) and insulin-like growth factor (IGF) [9, 10]. Once these factors bind to their corresponding receptors, Janus kinases (JAKs) are activated [11]. JAKs phosphorylate the cytoplasmic tail of the cognate receptor and STAT3 via its SH2 domain binds to phosphorylated tyrosine residues. The phosphorylated STAT3 forms homodimers and translocate into nucleus and, thus, can exchange signals between the cytoplasm and nucleus. Upon translocation into the nucleus, pSTAT3 forms a complex with some coactivators, including p68, and binds to the promotor region of target genes to activate their transcription [12]. This review aims to explore the mechanism of STAT3 in breast cancer development and summarize the latest advancements made.

## Advances in the study of STAT3 signaling pathways in breast cancer

### The role of STAT3 in breast cancer progression

**Fig. 2:**
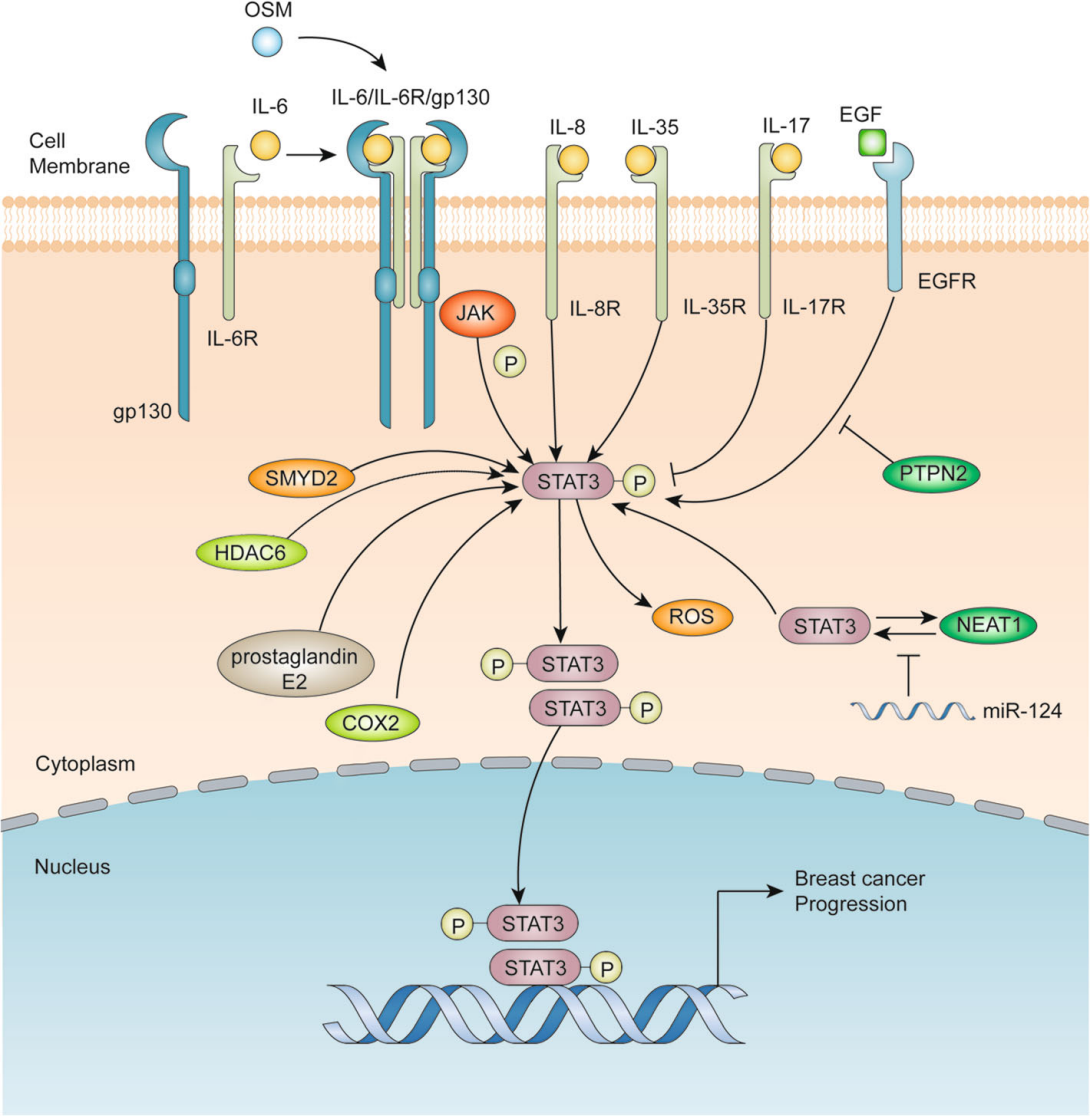
Advances of the STAT3 signaling pathways involved in breast cancer progression. Interleukins, including IL-6, IL-8 and IL-35, can bind to their receptors and activate the phosphorylation of JAKs and STAT3, OSM can increase IL-6-mediated activation, and IL-17 binding to its receptor leads to inhibition of STAT3 phosphorylation. STAT3 phosphorylated by EGF can be inhibited by PTPN2. COX2 and prostaglandin E2 upregulated by HDAC6 can activate STAT3 phosphorylation, and SMYD2 has a similar effect. Additionally, STAT3 and NEAT1 can form a loop to activate the phosphorylation of STAT3, which is inhibited by miR-124. The activated and phosphorylated STAT3 dimers translocate into the nucleus and activate the transcription of target genes involved in breast cancer progression

An illustration of the advances in our understanding of the STAT3 signaling pathways in breast cancer progression is shown in Fig. 2. A member of the IL-6 family of cytokines, Oncostatin M (OSM) can induce IL-6 upregulation and STAT3 phosphorylation to promote breast cancer progression [13] and to activate STAT3 and hypoxia inducible factor 1 alpha (HIF-1α) in estrogen receptor (ER)- breast cancer cells or in ER+ breast cancer cells in cooperation with IL-6 [14]. Additionally, other interleukins, such as IL-35 and IL-8, are also found to promote breast cancer progression by activating STAT3. IL-35 is found to inhibit conventional T (T-conv) cells and promote breast cancer progression via activation of STAT1 and STAT3 [15], whereas IL-8 and growth-regulated oncogene (GRO) chemokines are found to activate STAT3 and promote the progression of inflammatory breast cancer [16]. In contrast, low expression of IL-17 is found to inhibit STAT3 activation [17].

Other mediators of STAT3 expression and activation include activators, such as prostaglandin E2, cyclooxygenase-2 (COX2) and SET and MYND (myeloid-Nervy-DEAF-1) domain-containing protein (SMYD2), as well as its inhibitors, such as microRNA and protein tyrosine phosphatase 2 (PTPN2). Epigenetic regulators have been widely investigated and discovered to regulate STAT3 activation in breast cancers in recent years. Li et al. have found that histone deacetylase 6 (HDAC6), a class II histone deacetylase, and prostaglandin E2 and COX2, can upregulate STAT3 activation in breast cancer [18]. In addition, lysine methyltransferase SMYD2 can activate the methylation and phosphorylation of STAT3 to promote breast cancer progression [19]. MicroRNA (miR) has become a hot topic in the fields of cancer biology and development in recent years. Pang et al. have demonstrated that nuclear enriched abundant transcript 1 (NEAT1) forms a feedback loop with STAT3 to promote breast cancer progression. However, NEAT1 is suppressed by miR-124 [20]. Interestingly, glucosamine is found to suppress the activation of STAT3 and decrease breast cancer stemness and progression [21]. Additionally, knockdown of PTPN2 leads to EGF-mediated STAT3 activation [22]. The association of chronic inflammation with breast cancer progression is widely recognized, but it can be inhibited by blocking STAT3 [23]. Other mediators of STAT3 signaling pathways are also extensively studied. Kim et al. have found that the IL-6/STAT3/ROS pathway can not only promote breast cancer progression and inflammation but also increase the formation of breast cancer stem cells [24]. Moreover, TGFβ-regulated FAM3C/Interleukin-like EMT Inducer (ILEI), an oncogenic member of the FAM3 cytokine family, can mediate STAT3 signaling pathway to drive breast cancer stem cell formation and promote breast cancer progression [25]. In addition, TNFRSF1A, a gene encoding a transmembrane receptor for TNF-α, can be modulated by STAT3 and promote NF-κB signaling in breast cancer [26].

There were also some STAT3 co-factors influenced the proliferation and progress of breast cancer. Progranulin (PGRN), was seen to associate with chemoresistance and worse prognosis in breast cancer [27, 28], and the use of a specific progranulin antisense oligonucleotide was recently seen to hamper STAT3 oncogenic functions in CRC cells [29], suggesting a similar effect also in breast cancer cells. The cyclin dependent kinase 5 (CDK5) regulatory subunit-associated protein 3 (CDK5RAP3, also called C53/LZAP) was originally regarded as a p53 co-activator [30]. A recent research reported that CDK5RAP3 was associated with primary breast cancer progression and proliferation, and also enhanced the expression of STAT3-dependent genes [31]. Thus, targeting the co-factor of STAT3 maybe a potential therapeutic approach in breast cancer management.

### The role of STAT3 in breast cancer proliferation and apoptosis

**Fig. 3:**
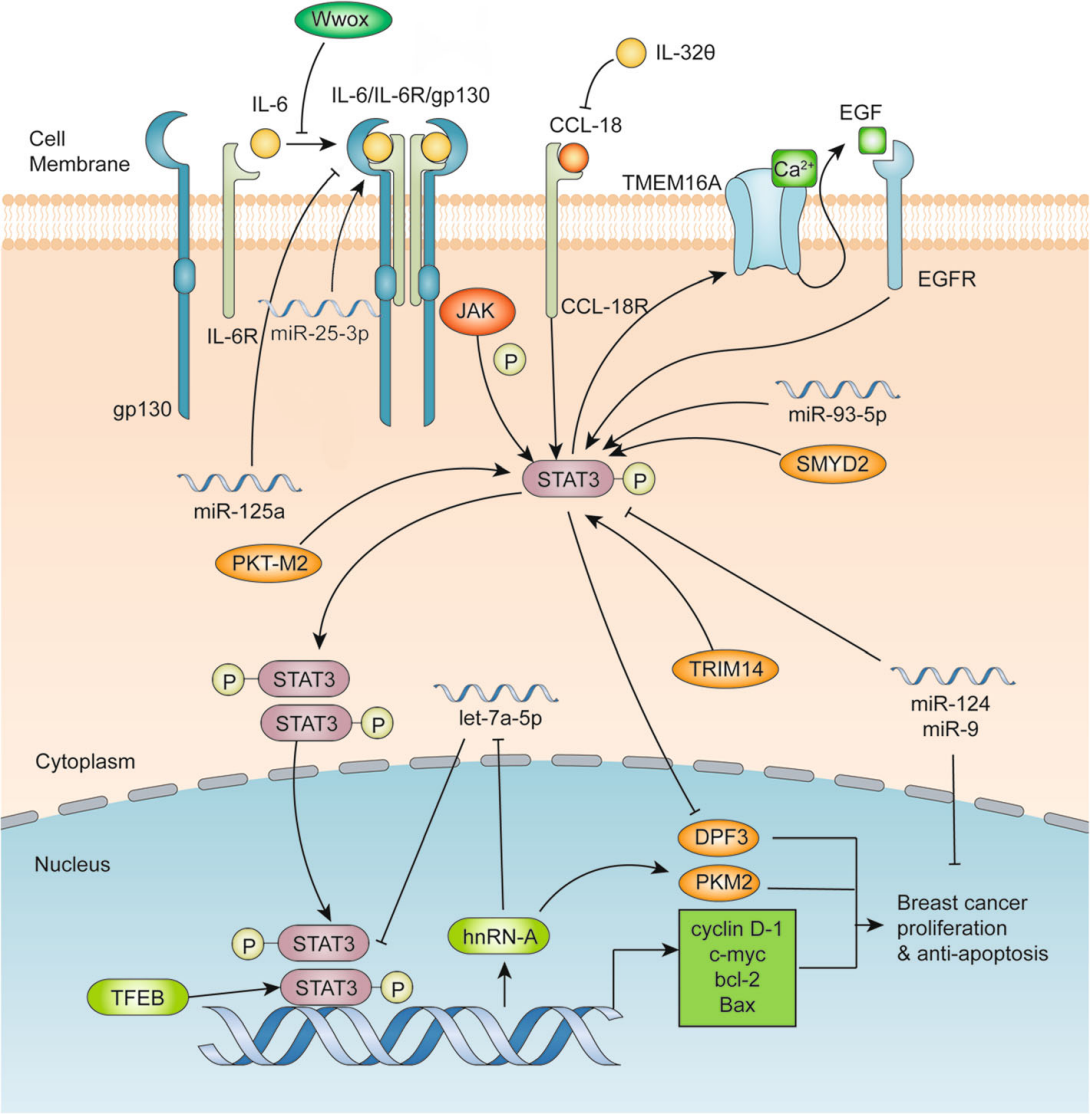
Advances of the STAT3 signaling pathways involving breast cancer proliferation and apoptosis. Classical IL-6 /JAK/STAT3 pathways can activate the transcription of cyclin D-1, c-myc, bcl-2 and Bax to promote the proliferation and inhibit the apoptosis of breast cancer. miR-125a, miR-25-3p and p16 can promote the binding of IL-6 to its receptors, whereas Wwox has the opposite effect. CCL-18 binding to its receptor can activate the phosphorylation of STAT3, which can be inhibited by IL-32θ. The circuit loop of phosphorylated STAT3, TMEM16A and EGF leads to continuous activation of STAT3. miR-93-5p, SMYD2, TRIM14 and PKT-M2 induce the activation of STAT3, whereas miR-124 and miR-9 inhibit the activation of STAT3 and breast cancer proliferation. Let-7a-5p, hnRN-A and phosphorylated STAT3 dimers form a circuit loop to upregulate PKM2 and promote the proliferation and inhibit the apoptosis of breast cancer cells. DPF3 suppressed by phosphorylated STAT3 can promote breast cancer proliferation. Additionally, transcription factor EB (TFEB) can combine with phosphorylated STAT3 dimers to promote the transcription of target genes involved in breast cancer proliferation

The illustration with advances of STAT3 signaling pathways in breast cancer proliferation and apoptosis is shown in Fig. 3. A recent research has reported that downregulation of zinc-finger gene DPF3 (also known as CERD4) promotes proliferation and motility of breast cancer via activating JAK2/STAT3 pathway [32]. It has been reported earlier that STAT3 can upregulate cyclin D-1, c-myc, and bcl-2 to suppress the apoptosis of breast cancer cells, indicating a potential involvement of STAT3 in cell cycle and survival [33]. Moreover, STAT3 activated by IL-6/JAK2 pathway can inhibit Bax/Bcl-2-related caspase-dependent apoptosis [34]. However, overexpression of WW domain-containing oxidoreductase (Wwox) blocks the combination of STAT3 and IL-6R, resulting in inhibition of proliferation [35]. Another research shows that IL-32θ targets chemokine ligand (CCL)18/STAT3 pathway to suppress macrophage-promoted breast cancer progression [36]. In addition, miRNAs are also widely investigated in breast cancer proliferation and invasion. Park et al. have found that miR-125a and let-7e could inhibit IL-6/STAT3 pathway to mediate the breast cancer proliferation and vasculogenic mimicry formation [37], and Shi et al. have found that miR-124 could suppress the mRNA and protein levels of STAT3 and inhibit the proliferation and invasion of breast cancer [38]. Similarly, miR-9 is reported to inhibit STAT3 activation and breast cancer proliferation [39]. In contrast, miR-93-5p and miR-25-3p are found to mediate STAT3 and promote breast cancer proliferation [40, 41]. Since the discovery of Warburg effects, metabolism is strongly linked with proliferation of cancer cells. It has been suggested that let-7a-5p, Stat3, and hnRNP-A1 form a feedback loop to regulate PKM2 expression and modulate glucose metabolism in breast cancer cells, suggesting that inhibiting STAT3-related metabolism may inhibit breast cancer proliferation [42].

There are several new pathways associated with STAT3 and breast cancer that have been minimally studied to date. It has been revealed that the Ca^2+^ activated chloride channel TMEM16A forms an activation loop with EGFR/STAT3 to promote breast cancer proliferation [43]. Moreover, tripartite motif-containing 14 (TRIM14) is found to increase the expression of p-STAT3 to promote breast cancer proliferation [44]. In addition, it is reported that pyruvate kinase type M2 (PKT-M2) regulates phosphorylation of STAT3 in breast cancer [45], whereas cystathionine-lyase (CSE) suppresses the expression of STAT3/matrix metallopeptidases-2 (MMP2), MMP9, p-protein kinase B and B-cell lymphoma 2 [46].

### The role of STAT3 in breast cancer metastasis

**Fig. 4:**
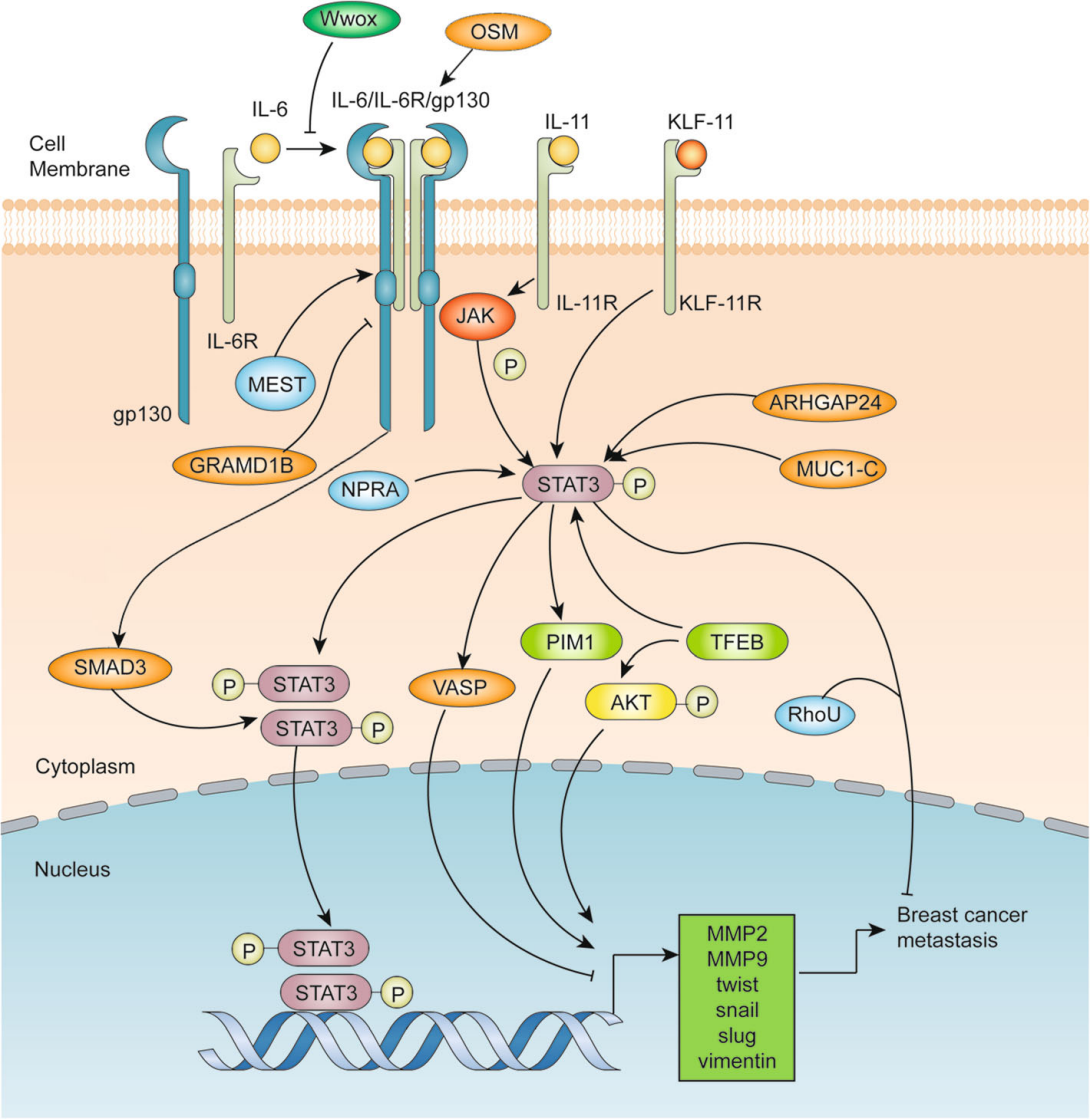
Advances of the STAT3 signaling pathways involving breast cancer metastasis. Classical IL-6/JAK/STAT3 pathways activate the transcription of MMP2, MMP9, Twist, Snail, Slug and vimentin to promote breast cancer metastasis, which can be suppressed by MEST and activated by GRAMD1B. Wwox can inhibit the binding of IL-6 and IL-6R/gp130. IL-11 and KLF-11 can also activate STAT3 to promote breast cancer metastasis by binding to their receptors. ARHGAP24, MUC1-C, NPRA and OSM-mediated SMAD3 function to upregulate the phosphorylation of STAT3. Estrogen related receptor alpha (ERR-α) can be transcriptionally activated by STAT3 and promote breast cancer metastasis. Phosphorylated STAT3 induces the activation of VASP to inhibit the metastasis of breast cancer, whereas PIM1 induced by phosphorylated STAT3 may have the opposite effect. The combination of phosphorylated STAT3 and RhoU inhibits breast cancer metastasis. Additionally, TFEB can activate the phosphorylation of STAT3 and AKT to promote breast cancer metastasis

An illustration of the advances of the STAT3 signaling pathways in breast cancer metastasis is shown in Fig. 4. Matrix metallopeptidases (MMPs) are known to play important roles in breast cancer metastasis. A well-studied mechanism of STAT3-mediated cell metastasis is through upregulating MMP2, MMP9, Twist, Snail, Slug and vimentin [47–49]. Ma et al. have reported that inhibition of STAT3 phosphorylation could reduce the expression of vasodilator-stimulated phosphoprotein (VASP), MMP2 and MMP9 in breast cancer [50]. As mentioned previously, STAT3 signaling is usually activated upon binding of cytokines and growth factors to their cognate receptors on the plasma membrane. The previously mentioned Wwox can inhibit breast cancer metastasis by preventing receptor binding [35]. Furthermore, Kim et al. have demonstrated that Mesoderm-specific transcript (MEST) induces Twist expression by activating the JAK/STAT3 signaling pathway [51], whereas Khanna et al. have shown the inhibition of GRAM domain-containing protein 1B (GRAMD1B) in breast cancer migration via the suppression of the JAK/STAT3 and protein kinase B (Akt) pathway [52]. Instead of classical ligand/receptor binding in the plasma membrane for STAT3 activation, a new pathway is found in which OSM/SMAD3 could also activate STAT3 and mediate Snail expression and promote epithelial-mesenchymal transition (EMT) in breast cancer, indicating a distinct route of STAT3 activation through cytoplasmic molecules and endogenous signaling [53]. Other signaling molecules, including miRNA, proto-oncogene serine/threonine-protein kinase (PIM1), Mucin-1-C (MUC1-C), natriuretic peptide receptor A (NPRA) and RhoU, were also discovered to participate in STAT3-mediated breast cancer metastasis. miR-30d is found to mediate migration and invasion in breast cancer cells by regulating Krüppel-like factor 11 (KLF-11), a new exogenous signaling pathway that can activate STAT3 by binding to its transmembrane receptor KLF-11R [54]. In addition, IL-11 is also found to regulate the JAK/STAT3 pathway in breast cancer-bone metastasis [55]. PIM1, a proto-oncogene responsible for promoting cell invasion and upregulating EMT expression in breast cancer, is found to be regulated by the IL-6/STAT3 signaling pathway [56]. MUC1-C, an oncogenic protein, can activate STAT3 and induce Twist transactivation to promote EMT [57]. Moreover, NPRA, one of the natriuretic peptide receptors, is found to increase the expression of STAT3 and MMP9 to promote the migration and invasion of breast cancer cells [58]. STAT3, by cooperating with Specificity Protein 1 (SP1), is found to induce high Ras Homolog Family Member U (RhoU) expression and breast cancer cell migration [59]. Additionally, some enzymes are also found to participate in breast cancer metastasis by the posttranscriptional modification of STAT3. ARHGAP24, a Rac-specific Rho GTPase-activating protein (Rho GAP), is found to promote phosphorylation of STAT3 and to increase the expression of MMP2 and MMP9 in breast cancer cells [60]. GCN5, a histone acetyltransferase, is found to upregulate the expression of p-STAT3, p-AKT, MMP9 and E2F1 and promote breast cancer migration and invasion [61].

Hypoxia is a stressed state that is extensively studied in cancers. Abyaneh et al. have found that hypoxia can significantly induce the activation of STAT3 to promote breast cancer stemness and metastasis [62]. This phenomenon provides us with a new direction for STAT3 research and targeted STAT3 therapy in breast cancer. Moreover, our recent research has found that estrogen related receptor alpha could promote the metastasis of triple negative breast cancer as a target gene of STAT3 [63].

### The role of STAT3 in breast cancer chemoresistance

**Fig. 5:**
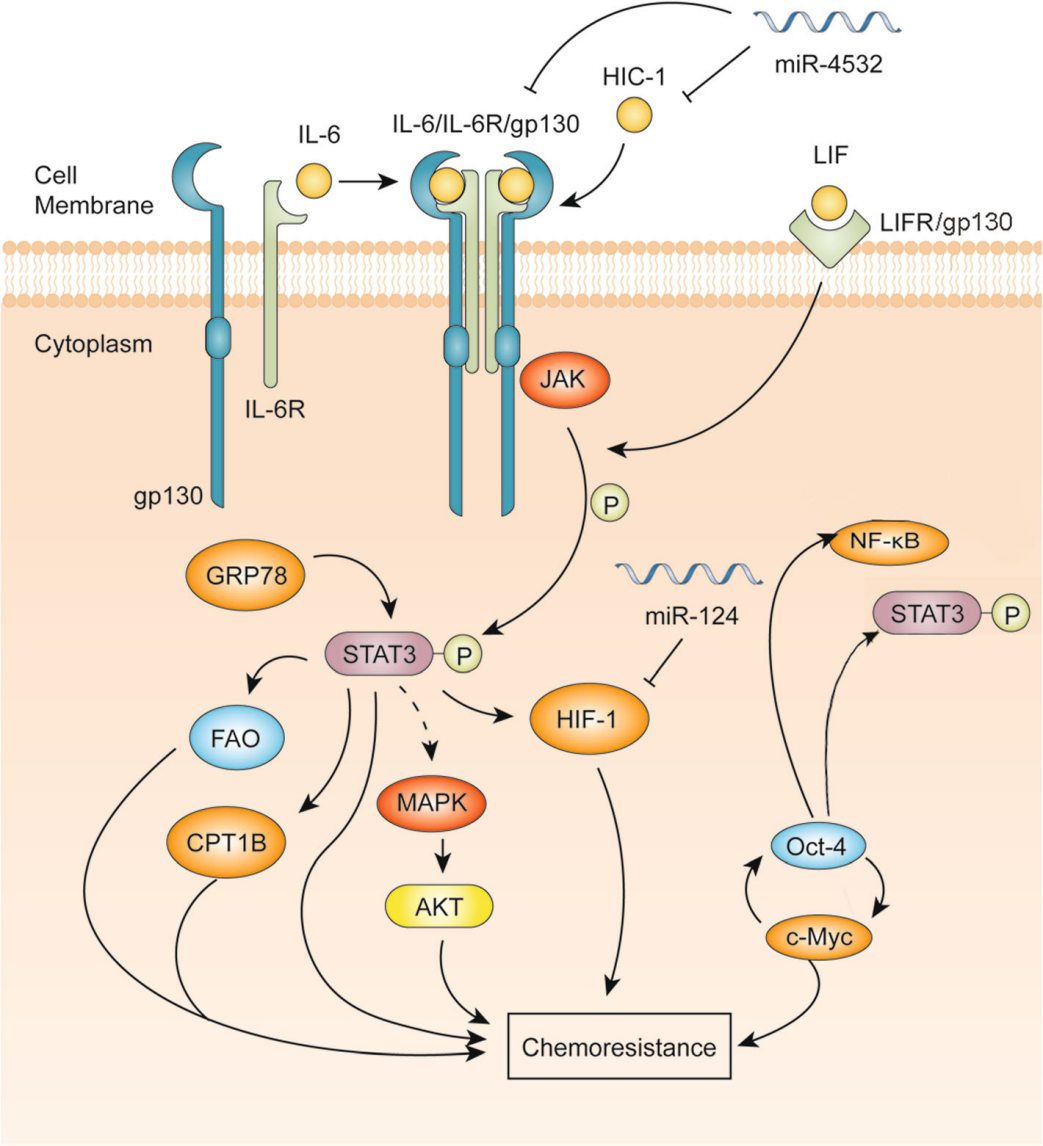
Advances of the STAT3 signaling pathways involving breast cancer chemoresistance. Classical IL-6/JAK/STAT3 pathways can induce chemoresistance in breast cancer, while miR-4532 may attenuate this effect by inhibiting HIC-1 and IL-6/STAT3 pathways. Leukemia inhibitory factor (LIF) binding to its receptor LIFR can increase the activation of STAT3. STAT3 and pSTAT3 levels are regulated by GRP78. Then, phosphorylated STAT3 activates cellular molecules including FAO, CPT1B and MAPK/AKT to induce the chemoresistance of breast cancer. Oct-4 and c-Myc form a signaling loop to promote STAT3/NF-κB activation and chemoresistance in breast cancer. Additionally, miR-124 can inhibit HIF-1 and promote breast cancer chemoresistance

An illustration of the advances of the STAT3 signaling pathways in breast cancer chemoresistance is shown in Fig. 5. Tzeng et al. have indicated that the Src/STAT3 signaling pathway is involved in multidrug resistance in triple negative breast cancer cells [64]. It is also found that crosstalk between breast cancer cells and macrophages can induce tamoxifen and ICI 182,780 resistance through the NF-κB/STAT3/ERK pathways [65].

The newly discovered downstream targets of STAT3-mediated chemoresistance include fatty acid beta-oxidation (FAO), carnitine palmitoyltransferase 1B (CPT1B), mitogen-activated protein kinase (MAPK)/AKT, HIF-1 and octamer-binding transcription factor-4 (Oct-4). It has been found that the JAK2/STAT3 signaling pathway increases CPT1B and FAO to increase chemoresistance in breast cancer [66]. Wang et al. found that IL-22 can promote JAK-STAT3/MAPKs/AKT pathway activation to induce breast cancer migration and paclitaxel resistance [67]. Moreover, miR-124 has been identified to reverse doxorubicin (DOX) resistance of breast cancer cells through targeting the STAT3/hypoxia-inducible factor 1 (HIF-1) pathway [68]. A recent study shows that Oct-4 and c-myc can form a signal circuit to increase Adriamycin resistance in breast cancer [69]. Meanwhile, Kim et al. have discovered that Oct-4 confers radiation resistance via STAT3 and NF-B-mediated IL-24 production in breast cancer cells [70]. In addition, paclitaxel is widely used as a clinical drug of breast cancer treatment, and phosphorylated STAT3 could mediate Survivin to promote paclitaxel resistance [71].

There are several upstream regulators of STAT3-mediated chemoresistance that have been identified in recent years. The COOH-terminal proline-rich region of 78-kDa glucose-regulated protein (GRP78), by regulating STAT3, is found to play a crucial role in the development of tamoxifen-resistant breast cancer cells [72]. Wang et al. have found that leukemia inhibitory factor receptor (LIFR) could promote STAT3 activation and contribute to breast cancer resistance to Trastuzumab-emtansine (T-DM1) [73]. Furthermore, miR-4532 is found to suppress hypermethylated in cancer-1 (HIC-1) and IL-6/STAT3 to promote Adriamycin resistance in breast cancer [74].

Some small molecules have also been found to contribute to chemoresistance mediated by STAT3. Piperlongumine combined with DOX is also found to induce apoptosis and inhibit DOX resistance of breast cancer cells via the JAK/STAT3 pathway [75]. In addition, targeting IL6/STAT3 activity using STAT3 inhibitor combined with a poly ADP-ribose polymerase (PARP) inhibitor could effectively treat palbociclib resistance in breast cancer cells [76].

## Advances in the study of compounds targeting STAT3 in breast cancer

### Compounds inhibiting the upstream of STAT3 in breast cancer

Several compounds are found to inhibit the upstream mediators of STAT3 in breast cancer since 2018 (Table 1). Many of these compounds target the IL-6/STAT3 signaling pathway. Ilamycin C is found to induce apoptosis and inhibit migration and invasion by suppressing the IL-6/STAT3 pathway [34]. A small molecule, bazedoxifene, is a novel IL-6/GP130 inhibitor that reduces breast cancer proliferation and migration [77]. Moreover, Esparza-Lopez et al. have discovered the inhibitory effect of metformin in IL-6-induced proliferation and EMT through the STAT3/NF-κB pathway in breast cancer [89]. DT-13, the saponin monomer 13 of the Dwarf lilyturf tuber, has been identified as a suppressor of breast cancer metastasis that acts by inhibiting both JAK/STAT3 and PI3K/AKT signaling pathways [81]. Furthermore, a natural compound called esculentoside A, a triterpene saponin derived from the root of Phytolacca esculenta, can also inhibit the IL-6/STAT3 pathway [78]. Meanwhile, another nature compound called catechol, which is derived from Aronia juice, shows similar effects in breast cancer cells [79]. In addition, scorpion venom can decrease IL-6, RhoC, ERK (1/2), and STAT3 and inhibit breast cancer proliferation [80]. As discussed previously, dihydrotanshinone inhibits breast cancer cells progression and stem cell formation through the IL-6/STAT3 pathway [24].

**Table 1:** Compounds inhibiting STAT3 in breast cancer since 2018

	Proposed Effects	Inhibitor	Cancer cell line tested	Refs
Signaling Pathways	Inhibiting IL-6/JAK/STAT3 pathway	Ilamycin C	MCF-7, MCF-10A	[34]
		bazedoxifene	SUM159, MDA-MB-231, MDA-MB-468	[77]
		esculentoside A	MCF-7, MCF-10A, LO2	[78]
		catechol	MCF-7, MDA-MB-231	[79]
		scorpion venom	HCT-8, MDA-MB-231	[80]
		dihydrotanshinone	MCF-7, MDA-MB-231	[24]
		DT-13	MDA-MB-231, MDA-MB-468	[81]
		ganoderic acid A	MDA-MB-231	[82]
		methylseleninic acid	4 T1	[83]
		sesquiterpenoid	MDA-MB-231	[84]
		sabutoclax	MCF-7	[85]
		tagalide A and tagalol A	MDA-MB-453, MDA- MB-231, SK-BR-3, MCF-7, MT-1, ZR-75-1	[86]
	Inhibiting SIRT1/STAT3 pathway	I157172	MCF-7	[43]
	Inhibiting miR-124/STAT3 pathway	cyanidin-3-glucoside	MDA-MB-231, Hs-578 T	[87]
	Inhibiting EGFR/STAT3/Akt pathway	CAPE-pNO_2_	MDA-MB-231	[88]
	Inhibiting STAT3/NF-κB pathway	metformin	MBCDF, MBCD3, MBCD4, MBCD17, MBCD23, MBCD25	[89]
		alantolactone	MDA-MB-231	[90]
	Inhibiting STAT3/Nanong pathway	isoharringtonine	HCC1806, HCC1937, MCF-7	[91]
Suppressing STAT3 function	Inhibiting STAT3 phosphorylation and dimerization	Galiellalactone SG-1709 SG-1721	BT-549, BT-20, MDA–MB-468, MCF-7, T47D, SK-BR-3, MDA–MB-453	[92]
	Inhibiting STAT3 phosphorylation/ activation	schisandrin A	MCF-7	[93]
		hexane fraction	MDA-MB-231	[33]
		ruxolitinib	MCF-7	[94]
		pyrimethamine	TUBO, TM40D-MB	[95]
		stattic	ZR-75-1	[96]
		niclosamide	MCF-7, MDA-MB-231, MDA-MB-468	[97]
		flubendazole	MDA-MB-231, Hs578T, BT-549, 4 T1	[98]
		eupalinolide J	HEK 293, MDA-MB-468, MDA-MB-231	[99]
		betulinic acid	MCF-7, MDA-MB-231	[100]
Direct binding to STAT3	Binding to SH2 domain	cryptotanshinone KYZ3	MDA-MB-231, MDA-MB-468, MCF-10A, L02	[101]
		napabucasin	MDA-MB-231	[102]
		coumarin-benzo [b] thiophene 1, 1-dioxide conjugates	MDA-MB-231, LO_2_, HepG2	[103]
	Binding to Cys 259 and 251 sites	15-keto PGE2	MCF10A, MDA-MB-231, PC3	[104]
	Others	risedronate sodium and zoledronic acid	MCF-7, MDA-MB-231	[105]
		osthole	MDA-MB-231, BT-549, MDA-MB-468, MCF-7	[106]

Other compounds target different signaling pathways, including the JAK2/STAT3 and Akt pathways. Both ganoderic acid A, which is isolated from ganoderma, and methylseleninic acid are found to suppress breast cancer proliferation via the JAK2/STAT3 pathway [82, 83]. A compound called caffeic acid p-nitro-phenethyl ester (CAPE-pNO_2_) is found to inhibit the EGFR/STAT3/Akt pathway and suppress breast cancer proliferation and metastasis [88]. Moreover, I157172, a novel inhibitor of cystathionine-lyase, is found to inhibit the proliferation and migration of breast cancer cells via upregulation of SIRT1 and inhibition of STAT3 signaling pathway [46].

Other compounds target the regulation of STAT3 expression. Alantolactone, a sesquiterpene lactone, can significantly decrease the expression of STAT3 and NF-κB in breast cancer [90]. Similarly, cyanidin-3-glucoside (C3G) can increase miR-124 expression and attenuate breast cancer proliferation by downregulating STAT3 expression [87].

### Compounds inhibiting the activation of STAT3 in breast cancer

In recent years, various novel compounds have been found to inhibit the phosphorylation and activation of STAT3. A sesquiterpenoid from *Farfarae Flos* (ECN) is found to inhibit the phosphorylation and dimerization of STAT3 in the JAK/STAT3 pathway [84]. Moreover, (−)-galiellalactone and its novel analogues, SG-1709 and SG-1721, are found to inhibit STAT3 phosphorylation and suppress the dimerization and DNA-binding of STAT3 in breast cancer [92]. Similarly, schisandrin A is found to reverse doxorubicin resistance via inhibition of STAT3 phosphorylation in breast cancer [93]. Chun et al. have found that the hexane fraction from *I. helenium* (HFIH) can inhibit STAT3 phosphorylation at tyrosine 705 [33]. Niclosamide, that was reported to be a potent STAT3 inhibitor in TNBC cells, was found to overcome the radioresistance in TNBC cells via inhibition of STAT3 and Bcl-2 activation and induction of reactive oxygen species (ROS) [97]. In addition, flubendazole (FLU), a widely used anthelmintic agent, eupalinolide J, a Michael-reaction acceptor extracted from *Eupatorium lindleyanum*, and betulinic acid are found to inhibit STAT3 activation in breast cancer cells [98–100]. As an upstream activator of STAT3, inhibition of JAK2 can undoubtedly suppress STAT3 activation. The classical JAK2 inhibitor is known as AG490. Recently, ruxolitinib is found to have a potential to be a new selective JAK2 inhibitor and to block STAT3 activation [94]. Furthermore, tagalide A and tagalol A are also found to inhibit the phosphorylation of STAT3 and JAK2 in breast cancer [86]. Additionally, sabutoclax, a pan-active BCL-2 protein family antagonist, is found to inhibit the IL-6/STAT3 pathway and thereby overcome multidrug resistance in breast cancer [85], whereas isoharringtonine (IHT) is found to suppress the STAT3/Nanong pathway to inhibit breast cancer proliferation [91].

Notably, some STAT3 inhibitors are found to function in many biological processes. Sravanthi et al. have screened 29,388 ligands docking with STAT3 and found that Risedronate Sodium (RES) and Zoledronic acid (ZOL) could tightly combine with STAT3 and show significant cytotoxicity in breast cancer cells [105]. Moreover, a new synthetic derivative of cryptotanshinone KYZ3 is found to directly bind to the SH2 domain of STAT3 and act as a new STAT3 inhibitor [101]. Napabucasin and its angularly anellated isomer could also combine with SH2 domain of STAT3 [102]. One of coumarin-benzo [b] thiophene 1, 1-dioxide conjugates, compound 7a, could also combine with SH2 domain of STAT3 [103]. 15-Keto prostaglandin E-2 could bind to the Cys 251 and Cys 259 sites of STAT3 protein to inhibit the migration and proliferation of breast cancer [104]. Furthermore, pyrimethamine, a classic anti-microbial drug, is found to be a new STAT3 inhibitor and shows strong anti-cancer effects [95]. In addition, osthole, via binding to STAT3 protein, is found to suppress STAT3 activity and inhibit breast cancer cells apoptosis [106], whereas another STAT3 inhibitor, stattic, is found to promote the Bax/Bcl-2-mediated apoptosis in breast cancer and to increase the therapeutic effects of doxorubicin [96].

## Conclusions

In summary, evidence discussed in this review highlights the potential value of discovering new biological and physiological mechanisms in breast cancer. STAT3 acts as a transcriptional activator in breast cancer, which regulates several target oncogenes and affects breast cancer progression, proliferation, apoptosis, metastasis and chemoresistance. It is intriguing that various upstream regulators and downstream target genes have been newly discovered, suggesting potential targets that can be used for breast cancer therapy. Among these pathways, circuit loops and network crosstalk are notable. Together with the development of neural networks, these phenomena remind us that signaling pathways may not be regulated only in sequential order, suggesting that findings regarding the feedback-loops and networks still need our continuous attention. Using Bayesian inference, a mathematic framework, researchers have found that combination therapy targeting mTOR and STAT3 may be the best therapeutic target in breast cancer [107]. There were also several efficient and available clinical trials targeting STAT3, which was recently reported by Qin et al. [108]. Notably, several new specific STAT3 inhibitors have been found in recent years. Structure optimization of these inhibitors for reduced cytotoxicity to normal tissues and higher stability may be an interesting direction for researchers. Treatment with STAT3 inhibitors alone or combined with other clinical therapeutic drugs may provide more promising effects on suppressing or reversing chemoresistance in breast cancer. Especially for breast cancer patients suffering from doxorubicin or capecitabine resistance, STAT3 inhibitors instead of expensive monoclonal antibodies may be more beneficial. Therefore, STAT3 remains to be a strong clinical target for breast cancer prevention and therapy, which is worth continuous research.

## Data Availability

Not applicable

## Abbreviations

AKT: Protein kinase B
CCL: Chemokine ligand
COX: Cyclooxygenase
CPT: Carnitine palmitoyltransferase
EGF: Epidermal growth factor
FAO: Fatty acid beta-oxidation
FGF: Fibroblast growth factor
GRAMD: GRAM domain-containing protein
GRP: Glucose-regulated protein
HIF: Hypoxia inducible factor
IGF: Insulin-like growth factor
IL: Interleukin
JAK: Janus kinase
KLF: Krüppel-like factor
LIF: Leukemia inhibitory factor
MAPK: Mitogen-activated protein kinase
MEST: Mesoderm-specific transcript
MMP: Matrix metallopeptidases
MUC: Mucin-1-C
NEAT: Nuclear enriched abundant transcript
NPRA: Natriuretic peptide receptor A
OSM: Oncostatin M
PTPN: Protein tyrosine phosphatase
ROS: Reactive oxygen species
SMYD: SET and MYND ( myeloid-Nervy-DEAF-1) domain-containing protein
STAT: Signal transducer and activator of transcription
TFEB: Transcription factor EB
VASP: Vasodilator-stimulated phosphoprotein
